# Across-ear stimulus-specific adaptation in the auditory cortex

**DOI:** 10.3389/fncir.2014.00089

**Published:** 2014-07-30

**Authors:** Xinxiu Xu, Xiongjie Yu, Jufang He, Israel Nelken

**Affiliations:** ^1^Institute of Biophysics, Chinese Academy of SciencesBeijing, China; ^2^University of Chinese Academy of SciencesBeijing, China; ^3^Department of Neuroscience, Baylor College of MedicineHouston, TX, USA; ^4^Department of Biomedical Sciences, City University of Hong KongHong Kong, China; ^5^The Edmond and Lily Safra Center for Brain Sciences and the Department of Neurobiology, The Alexander Silberman Institute of Life Sciences, Hebrew UniversityJerusalem, Israel

**Keywords:** auditory cortex, rat, binaural interaction, sound location, stimulus-specific adaptation

## Abstract

The ability to detect unexpected or deviant events in natural scenes is critical for survival. In the auditory system, neurons from the midbrain to cortex adapt quickly to repeated stimuli but this adaptation does not fully generalize to other rare stimuli, a phenomenon called stimulus-specific adaptation (SSA). Most studies of SSA were conducted with pure tones of different frequencies, and it is by now well-established that SSA to tone frequency is strong and robust in auditory cortex. Here we tested SSA in the auditory cortex to the ear of stimulation using broadband noise. We show that cortical neurons adapt specifically to the ear of stimulation, and that the contrast between the responses to stimulation of the same ear when rare and when common depends on the binaural interaction class of the neurons.

## Introduction

In nature, unexpected or deviant stimuli may indicate events with important behavioral consequences. Thus, deviance detection is a vital task for the brain. At the single neuron level, deviance detection may be reflected in stimulus-specific adaptation (SSA), in which neurons respond more strongly to rarely presented stimulus than to the same stimulus when it is common (Ulanovsky et al., 2003; Antunes et al., 2010; Taaseh et al., 2011; Zhao et al., 2011; Nelken et al., 2013). In the auditory system, SSA has been identified in the midbrain, thalamus, and auditory cortex (Ulanovsky et al., 2003; Pérez-González et al., 2005; Gutfreund and Knudsen, 2006; Malmierca et al., 2009; von der Behrens et al., 2009; Yu et al., 2009; Antunes et al., 2010; Reches et al., 2010; Zhao et al., 2011). In those studies, neurons were tested with two different frequencies. Only two studies tested SSA for tone intensity, with inconclusive results (Ulanovsky et al., 2003; Farley et al., 2010), and there are reports of SSA for broadband stimuli (Nelken et al., 2013).

Interaural level difference (ILD) is an important feature of sounds, encoding to a large degree the azimuth of the sound source. It is initially computed in the superior olive, and further refined in higher brain centers. In the auditory cortex, neurons of different binaural interaction classes were reported to be topographically segregated independently of the isofrequency contours (Imig and Adrian, 1977; Middlebrooks et al., 1980; Reale and Kettner, 1986). In the present report, we examined whether SSA exists in the auditory cortex when tested with two different aural configurations in oddball sequences.

## Materials and methods

### Electrophysiology and stimulus presentation

Wister rat of both sexes, weighing 280–360 g with clean external ears served as subjects. Anesthesia was initially induced with sodium pentobarbital (60 mg/kg, 20% solution, ip, Sinopharm Chemical Reagent Co., Shanghai) and maintained by supplemental doses of the same anesthetic (20 mg/kg/h) during the surgical preparation and recording. Atropine sulfate (0.05 mg/kg, sc) was given 15 min before anesthesia to inhibit tracheal secretion. The preparation of the animal was a combination techniques described previously (He, 2003; Guo et al., 2007; Yu et al., 2008). Briefly, the subject was mounted in a stereotaxic device following the induction of anesthesia. A craniotomy was performed over the left auditory cortex. After the dura mater was removed a thin layer of warmed silicone oil was applied to the cortical surface to prevent desiccation. Throughout the experiment, the rat was kept on a heating blanket and body temperature was maintained at 37–38°C. The procedures were approved by the Animal Subjects Ethics Sub-Committee of the Institute of Biophysics, the Chinese Academic of Sciences.

The subject was placed in a double-walled soundproof room. The stimuli were broadband noise (bandwidth 2–40 kHz, 100 ms duration, 5 ms rise/fall times) which was generated digitally by an Auditory Workstation (Tucker-Davis Technologies, TDT, Alachua, FL) and were delivered dichotically to the subject via a coupled electrostatic speaker (EC1, TDT) mounted in a probe. Stimulus intensity was set to 65 dB (same sound level to both ears).

When a unit/multi-unit was found, the binaural response type was determined by pseudo-randomly presenting stimuli to ipsilateral ear, contralateral ear and both ears 20 times respectively (Kitzes et al., 1980; Kelly and Sally, 1988). In the oddball procedure, a train of noise bursts was presented monaurally. Standard/deviant stimuli were delivered directly to the ipsilateral/contralateral ear respectively in one block and the role of the two ears was switched in the other block (Ulanovsky et al., 2003; Yu et al., 2009). The probability of the deviant sounds was 10%, and they were presented in a periodic sequence of nine standard stimulus followed by a single deviant stimulus. The inter-stimulus interval (ISI, onset to onset) was 1000 ms.

### Recording and data analysis

Tungsten microelectrodes with impedances of 4–5 MΩ (Frederick Haer & Co., Bowdoinham, ME) were advanced by a stepping-motor microdrive. The depth range of recordings in the auditory cortex was from 700 to 1200 μm. The signal picked up by the microelectrode together with the acoustic stimulus signal was amplified and stored in the PC computer using TDT software (OpenEX, TDT) and Axoscope software (Axon Instruments, Sunnyvale, CA). The extraction of times of spike occurrence relative to stimulus delivery and all the other data analyses reported here were performed with Matlab software (Mathworks, Inc, Natick, MA).

Responses were quantified by calculating spike counts in a window of 50 ms, starting at the stimulus onset. PSTHs were calculated from over 180 trials for the standard and 20 trials for the deviant (for some units, 270 and 30 trials respectively) and smoothed only for display. We defined normalized stimulus-specific adaptation indices, SI (Ulanovsky et al., 2003), for each location as SI(ipsi) = [d(ipsi) – s(ipsi)]/[d(ipsi) + s(ipsi)], and SI(contra) = [d(contra) – s(contra)]/[d(contra) + s(contra)], where d(ipsi) and s(ipsi) were the responses to the ipsilateral ear when it was deviant and standard respectively, while d(contra) and s(contra) were the responses to the contralateral ear when it was deviant and standard respectively. The common SI (CSI) was computed similarly as the contrast between the sum of the two standard responses and the sum of the two deviant responses.

To classify the responses recorded from each unit, a three-letter code used in previous studies was employed with the first two letters expressing contralateral and ipsilateral responses respectively and the third the binaural interaction (Goldberg and Brown, 1969; Reale and Kettner, 1986; Kelly and Judge, 1994; Irvine et al., 1996). Neurons were classified as EE, EO, OE and PB (predominantly binaural, neurons that did not respond to stimulation of either ear, or only very weakly, but responded strongly to binaural stimulation). E represents the presence of an excitatory response to the corresponding ear while O represents poor or no response. Similar to earlier studies (Zhang et al., 2004), a criterion level of a 20% change in response was used to define the type of binaural interaction though it seems arbitrary. For EE neurons, the binaural interaction was classified as inhibition (I) if the binaural response was less than 80% of the monaural response to the dominant ear. It was classified as occlusion (O) if the binaural response was between 80% of the sum of the two monaural responses and 80% of the monaural response to the dominant ear. A binaural response that was within 20% of the sum of the respective monaural responses was considered to be no significant binaural interaction (N). A binaural interaction that produced a response to a binaural stimulus that was >120% of the sum of the monaural responses was classified as facilitation (F). For EO or OE neurons, inhibition (I) was considered to be present when the binaural response was less than 80% of the response to monaural stimulation of the dominant ear. The dominant ear was either contralateral ear for EO neurons or ipsilateral ear for OE neurons. A binaural response within ±20% of the response of the dominant ear was considered as indicative of no interaction (N). A binaural response greater than 120% of the monaural response to the dominant ear was classified as facilitation (F). We defined the binaural interaction index (BII) as the ratio of the binaural response to the contralateral response of EO neurons. The subclasses as defined here were somewhat arbitrary since a 20% increase or decrease could be statistically significant or not, and since the degree of binaural effects varied continuously over a continuum (e.g., Figure 4). However, the classification is still useful since it allows comparison with previous studies, and since it correlated with other features of the neuronal responses studied here, notably the SI.

**Figure 1:**
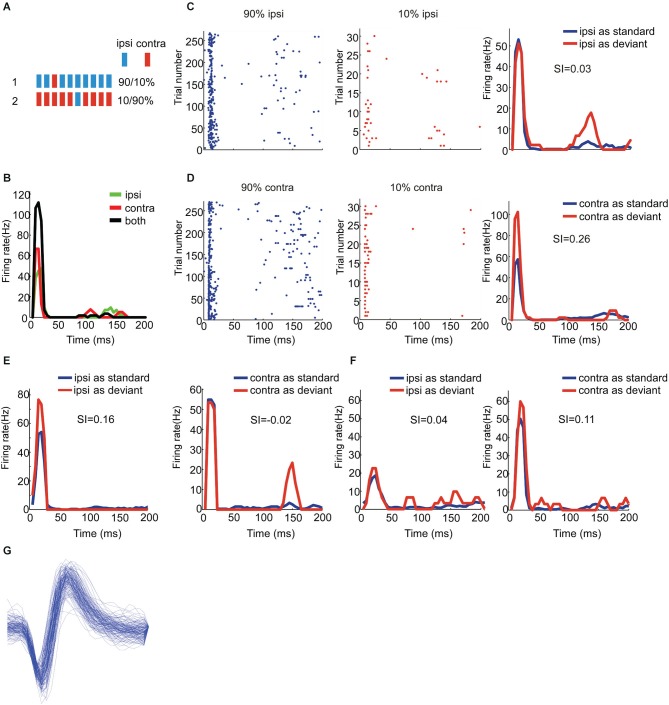
**Responses of EE neurons to stimuli with two locations presented in oddball procedure. (A)** Schematic of oddball procedure. The blue and red bars represent for stimuli delivered directly to ipsilateral and contralateral ear respectively. **(B)** Peristimulus time histograms showing one EE/N neuron’s monaural and binaural responses respectively. **(C, D)** Raster displays showing its responses to 270 times of standard (90% appearing probability, the left panel) and 30 times of deviant (10% appearing probability, the middle panel) stimuli presented to the ipsilateral and contralateral ear respectively; the right panels are the PSTH shown in the left and the middle. The stimulus duration and ISI was 0.1 and 1 s respectively. **(E, F)** Another two examples of EE/O and EE/I neuron respectively. **(G)** The spike waveform of a well separated neuron showing in panel **(E)**.

**Figure 2:**
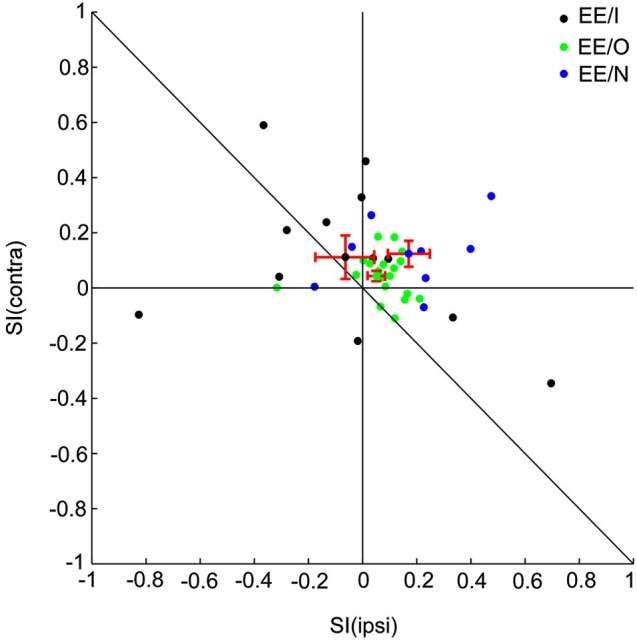
**Population analysis of SSA for all the EE neurons.** Scatter plot of SI (contra) vs. SI (ipsi) of EE/I (black), EE/O (green) and EE/N (blue) neurons.

**Figure 3:**
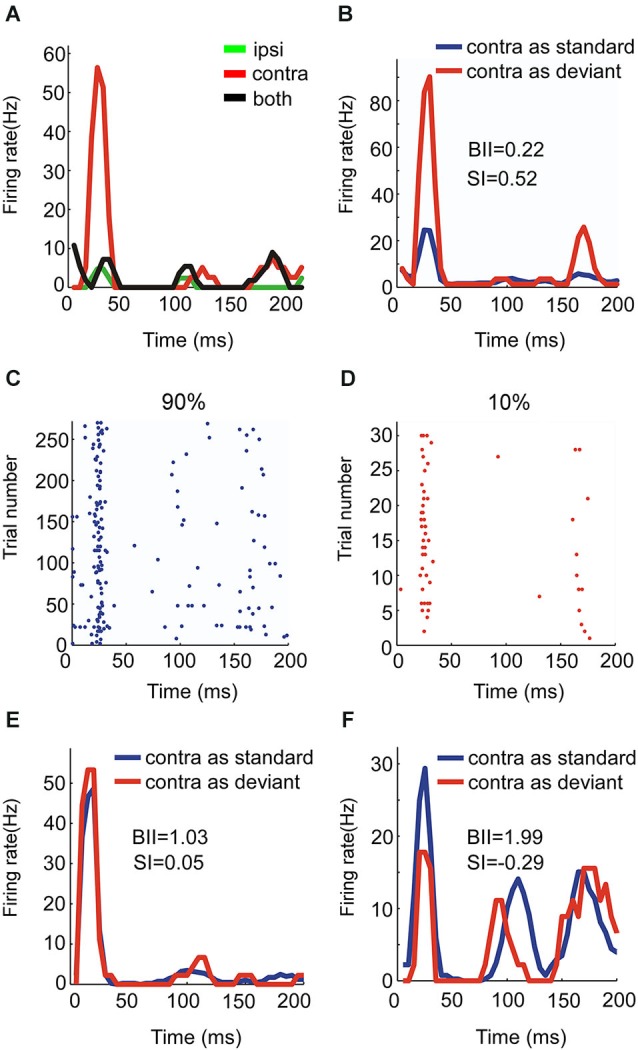
**Responses of EO neurons to noise burst with two locations presented in oddball procedure. (A)** Peristimulus time histograms showing one neuron’s monaural and binaural responses respectively. **(C, D)** Raster displays showing its responses to 270 times of standard (90% appearing probability) and 30 times of deviant (10% appearing probability) stimuli presented to the contralateral ear. **(B)** PSTH of responses shown in (**C** and **D**). **(E, F)** Another two examples of EO neurons with BII and SI (contra).

**Figure 4:**
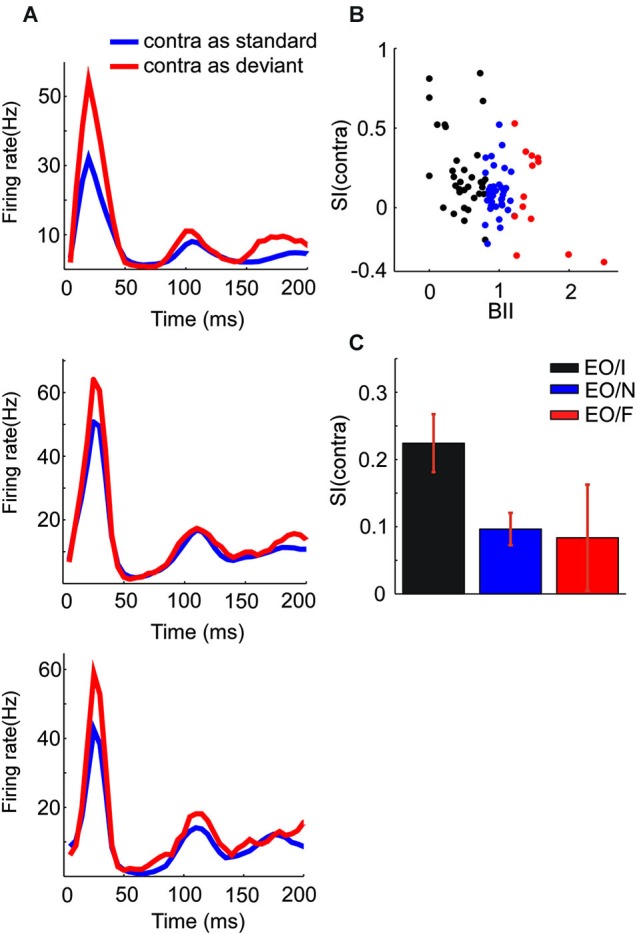
**Population responses of subgroups of EO neurons. (A)** PSTH showing the mean responses of EO/I (top), EO/N (middle) and EO/F (bottom) neurons in the oddball paradigm when presented to the contralateral ear. **(B)** The scatter plots the SI of three kinds of EO neurons vs. BII (binaural interaction index). There are three dots of which *x* value equaling 0 because they did not respond at all when the stimuli were presented to binaural ears. **(C)** Average SI of the classes of EO neurons (mean ± s.e.m., 0.224 ± 0.043, *p* < 10^−5^; 0.097 ± 0.024, *p* < 10^−3^; 0.084 ± 0.079, *p* = 0.312 for EO/I, EO/N and EO/F respectively, *t*-test).

## Results

### Classification of binaural neurons

**Figure 5:**
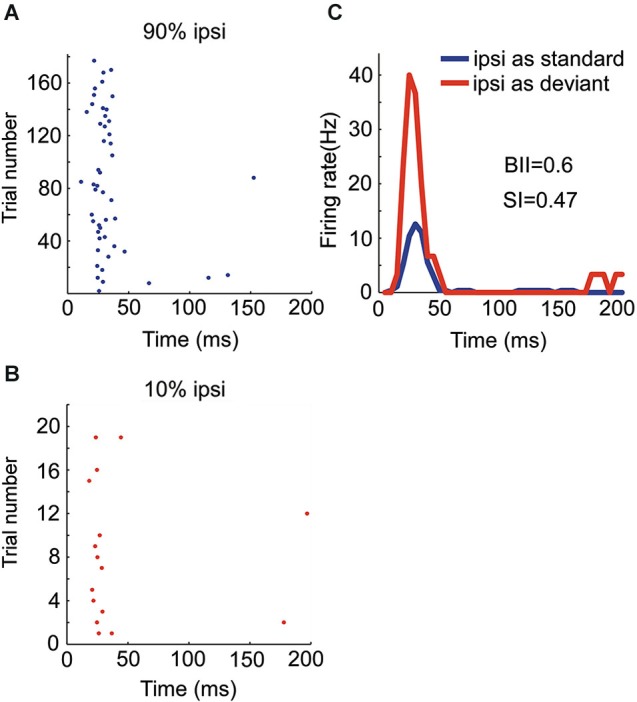
**Responses of an OE/I neuron to stimuli with two locations presented in oddball procedure. (A, B)** Raster displays showing its responses to 180 times of standard (90% appearing probability) and 20 times of deviant (10% appearing probability) stimuli presented to the ipsilateral ear. **(C)** PSTH of responses shown in (**A** and **B**).

The 129 single/multi-units recorded in the auditory cortex were divided into four groups according to their monaural response. The two largest groups were the EE group (39 neurons, 30%) that was monaurally excited by either ear and the EO group (84 neurons, 65%) that was monaurally excited only by the contralateral ear and had very weak or no response to the ipsilateral ear (see the Table 1). Only four neurons responded to the ipsilateral ear but not to the contralateral ear (OE). Two neurons were not excited by either ear alone but well excited when the stimulus delivered to both ears ipsilaterally and contralaterally (PB). Based on their binaural interactions, EE, EO and OE neurons were subdivided further. In the EE group, 31% (12/39) neurons were classified as EE/I, while 49% (19/39) neurons and 20% (8/39) neurons showed occlusive and no interactions respectively. Among the EO neurons, 44% (37/84) were classified as EO/N and 40% (34/84) as EO/I, with the rest (16%) classified as EO/F. Figure 1 shows responses of EE neurons, Figures 3 and 4 of EO neurons, and Figure 5 of an OE neuron.

**Table 1:** **Proportions of auditory cortical neurons showing different binaural interaction types**.

**Binaural Interaction**	***n***	**Proportion(%)**
EE	39	30
EE/I	12	9
EE/O	19	15
EE/N	8	6
EO	84	65
EO/I	34	26
EO/N	37	29
EO/F	13	10
OE	4	3
OE/I	2	1
OE/N	1	1
OE/F	1	1
PB	2	2

### SSA for aural configuration

We explored the presence of SSA to aural configuration in rat auditory cortex. We therefore used oddball sequences composed of two monaural stimuli: one was presented to the ipsilateral ear only, and the other to the contralateral ear only. Each unit was tested in two conditions. In the first, the standard consisted of monaural stimulation of the ipsilateral ear while the deviant consisted of monaural stimulation of the contralateral ear. In the second condition, the two stimuli were swapped (Figure 1A). In both conditions, deviants were presented periodically as every 10th stimulus in the block, and the inter-stimulus interval was always 1000 ms. All stimuli consisted of broadband noise.

We compared the responses of neurons to the same stimulus but with different probability of appearance. Figure 1 shows the responses of three EE neurons (panel **C** and **D** for EE/N, panel **E** for EE/O and panel **F** for EE/I). The EE/N neuron in Figures 1C,D had stronger responses to the contralateral monaural stimuli when they were presented as deviant than when they were presented as standard (Figure 1D). In other words, the neuron specifically adapted to the contralateral stimulus when standard. In contrast, its response to the ipsilateral stimulus did not depend on whether it was standard or deviant (Figure 1C). The EE/O neuron in Figure 1E, in contrast, had robust SSA to the ipsilateral stimulus but not to the contralateral stimulus. The EE/I neuron in Figure 1F had weak SSA to both aural configurations.

To quantify the degree of SSA, we adopted the stimulus-specific adaptation index (SI) for each aural configuration. The range of the index is between −1 and 1, and positive SI means stronger response to the deviant than to the standard. We plotted SSA (ipsi) vs. SSA (contra) using different colors to identify the different types of binaural interactions (Figure 2: EE/I, black; EE/O, green; EE/N, blue). For most of the EE neurons, the SI values were found above the reverse diagonal, indicating the presence of SSA (Ulanovsky et al., 2003). For the EE neurons, the CSI for the two auralities was significantly larger than 0 (*t* = 3.98, df = 38, *p* = 3*10^−4^). The CSI was not significantly different between the three classes of EE neurons (one-way ANOVA, *F*_(2,36)_ = 1.43, *p* > 0.05). The SI for the two aural configurations were not significantly different from each other on average (paired *t*-test, *t* = −0.76, df = 38, *p* > 0.05). Finally, there was no significant effect of group on either SI(contra) or SI(ipsi) (one-way ANOVA, for contralateral stimulation: *F*_(2,36)_ = 0.9, *p* > 0.05, for ipsilateral stimulation: *F*_(2,36)_ = 2.12, *p* > 0.05). Thus, the average SI was essentially the same for the ipsilateral and for the contralateral ears, and did not depend significantly on the binaural interaction subgroup.

EO neurons had a significant response to the contralateral ear only. Figure 3A show the responses of one EO neuron to the three aural configuration, showing a strong suppression of the contralateral response when both ears were stimulated. This neuron was therefore classified as EO/I. This neuron had a substantial SI to contralateral ear stimulation (Figure 3B). Raster plots of the responses of this neuron to contralateral stimulation when standard and when deviant are shown in Figures 3C,D. The EO/N neuron in Figure 3E showed an intermediate level of SSA. The EO/F neuron in Figure 3F had a stronger response to the standard than to the deviant stimuli, leading to a negative SI.

Across the entire EO population, the mean SI for the contralateral stimulus was significantly positive (*t* = 5.97, df = 83, *p* = 5*10^−8^). There were significant differences between the mean SI of the three subgroups of EO neurons (*F*_(2,81)_ = 3.7, *p* = 0.029). *Post hoc* comparisons (*p* = 0.05) showed that the EO/I neurons had on average a significantly larger SI(contra) than the EO/N neurons. The EO/F neurons had an average SI that was not significantly different from either of the two other groups, although this could be due to their smaller number. Figure 4 shows the mean population responses of the three kinds of EO neurons (panel **A**: top, EO/I; middle, EO/N; bottom, EO/F) and their mean SI (Figure 4C, 0.224 ± 0.043, 0.097 ± 0.024, 0.084 ± 0.079 for EO/I, EO/N and EO/F respectively, mean ± s.e.m.).

In fact, the degree of SSA and the binaural interaction of the EO neurons seemed to be continuously related to each other. Figure 4B plots the SI(contra) of EO neurons against their BII (see Section Materials and Methods). Small (close to 0) BII indicates a stronger suppression of the contralateral response by ipsilateral stimulation. There was a significant correlation between SI and BII (*F*_(1,82)_ = 14.0, *p* = 0.0003) for the EO neurons.

There were four OE neurons recorded in all. Among them, the neuron with the strongest SSA belonged to the OE/I class (Figure 5) with small BII equaling 0.6, consistent with the tendency of small BII to be associated with larger SI in the EO neurons. The mean SI (ipsi) for all the OE neurons was 0.197.

## Discussion

In this study, we found that neurons in auditory cortex show adaptation of their responses to noise bursts in an aurality-specific manner. EE neurons showed weak, but significant SSA to both ears, while EO neurons had SSA that depended on their binaural interaction class—the more suppressive the ipsilateral ear was on the responses to contralateral stimulation, the higher the SI (contra) tended to be. The few OE neurons behaved consistently with the same trend.

In the present study, multi-unit activity (MUA) was included based on the following considerations. As a rule, if the units composing the multiunit cluster were reasonably homogeneous, SSA from MUA was indicative of SSA at the single unit level. Confounding effects may have occurred when the selectivity of units to the different stimuli was different. For example, if an EO and an OE neurons were part of the same cluster, the cluster may have been classified as EE, producing an apparent SSA simply because one of the two units was adapted and the other was not to each single ear stimulation. However, such effects were unlikely here. First, units of the same binaural interaction type tend to be clustered in the auditory cortex (Kelly and Sally, 1988; Imig et al., 1990; Rajan et al., 1990; Clarey et al., 1994; Nakamoto et al., 2004), and anyway OE neurons are rare (see the Table 1 for the current dataset; these numbers are typical). Second, when MUA and single unit recordings have been compared in cortex, the response patterns have been found to be reasonably similar (for example, Taaseh et al., 2011 and Hershenhoren et al., 2014—the first used local field potential and MUA recordings, the second intracellular recordings; the two studies reported essentially the same level of SSA for tone frequency).

Our binaural interaction classes were of comparable distribution to previous reports. Compared to (Zhang et al., 2004), the percentage of EE neurons was similar (30% vs. 28.32%), but there was a slight increase in the proportion of EO neurons (65.12% vs. 50.44%). There were also minor differences in the classification of the subgroups, although these were probably due to the use of only one sound pressure level when testing the binaural interactions in our study, rather than varying the ILD (as in Zhang et al., 2004).

The size of SSA that we found was generally somewhat small compared to other reports in rat auditory system. However, we used stimuli that were somewhat longer (100 ms) than those used in many studies (30 ms: Malmierca et al., 2009; Taaseh et al., 2011, 70 ms: Duque et al., 2012; Hershenhoren et al., 2014), and the interstimulus interval we used (1000 ms) was longer than that used to elicit strong SSA (300–700 ms). In fact, von der Behrens et al. (2009), working in the awake rat, measured similar level of SSA using the same, relatively long, inter-stimulus interval. We conclude that the amount of SSA reported here is compatible with previous reports.

Although the present study was not intended to answer the question of the origin of cortical SSA, it is likely an accumulative effect partially inherited from the ascending pathway.

It is tempting to speculate on the mechanisms underlying these effects. To create EE neurons, inputs from the two ears need to be integrated by a single neuron on the path from the auditory nerve to the auditory cortex. Although EE neurons are present as early as the medial superior olive (MSO), binaural integration can occur at all succeeding stations—the inferior colliculus, auditory thalamus, and in auditory cortex itself. The SSA documented here in EE neurons is compatible with a model in which each of the monaural inputs to the binaural integration stage “fatigues” when used as the standard, and the SSA is the consequence of the other input being activated much more sparsely and therefore being less adapted. Such a model is essentially the ANTM (Adaptation in Narrowly Tuned Modules) model as described previously (Mill et al., 2011; Taaseh et al., 2011; Hershenhoren et al., 2014; Nelken, 2014), applied to binaural interactions. In fact, we predict that EE neurons, in any part of the auditory system, would show some form of SSA when tested with monaural oddball sequences of the type used here, and the cortical effects we observe could well be inherited from lower stations of the auditory system.

One trivial mechanism that may underlie the stronger SSA shown by EO (and OE) neurons could be also based on the fatigue of the excitatory input. This input is activated often when the dominant ear is standard, but rarely when it is deviant, and its differential adaptation state in the two conditions may give rise to the positive SI that we generally observed in such neurons. However, the results reported here actually suggest that the mechanisms shaping the SSA of EO neurons are more involved. The reason is the relationships between the strength of SSA these neurons had and the type of binaural interaction they showed. The stronger the inhibition of the non-dominant ear on the responses to the dominant ear was, the larger the SI tended to be, while the EO/N and EO/F subclasses actually showed very small SI and even negative SI (Figure 3). This suggests that the presumed inputs that are activated by the non-dominant ear on the EO/I neurons when the non-dominant ear is standard, while not evoking a response, do shape the responses to the rare presentations of the sound to the dominant ear. For example, in EO/I neurons, when the ipsilateral ear is standard, the neuronal excitability may increase due to post-inhibitory rebounds, resulting in the larger responses to the deviant sound presentations to the dominant ear.

It has been previously suggested that EI neurons (presumably corresponding mostly to our EO/I neurons) are related to sound localization while EE neurons are related to object detection (Manabe et al., 1978; Middlebrooks et al., 1980). Our use of the oddball sequences, and in particular the stronger SSA we found in EO/I neurons than in EE neurons, reinforces the link between EO/I neurons and the processing of azimuth information in auditory cortex.

## Conflict of interest statement

The authors declare that the research was conducted in the absence of any commercial or financial relationships that could be construed as a potential conflict of interest.
